# Understanding the Light Soaking Effects in Inverted Organic Solar Cells Functionalized with Conjugated Macroelectrolyte Electron‐Collecting Interlayers

**DOI:** 10.1002/advs.201500245

**Published:** 2015-12-16

**Authors:** Weidong Xu, Ruidong Xia, Tengling Ye, Li Zhao, Zhipeng Kan, Yang Mei, Congfei Yan, Xin‐Wen Zhang, Wen‐Yong Lai, Panagiotis E. Keivanidis, Wei Huang

**Affiliations:** ^1^ Key Laboratory for Organic Electronics and Information Displays (KLOEID) and Institute of Advanced Materials (IAM)Jiangsu National Synergetic Innovation Center for Advanced Materials (SICAM)Nanjing University of Posts and Telecommunications9 Wenyuan RoadNanjing210023China; ^2^ Department of Mechanical Engineering and Materials Science and EngineeringCyprus University of TechnologyDorothea Bldg 51145 Kitiou Kyprianou StreetLimassol3041Cyprus; ^3^ Department of ChemistryHarbin Institute of TechnologyHarbin150001China; ^4^ Center for Nano Science and Technology@PoliMiIstituto Italiano di TecnologliaVia G. Pascoli 70/3I‐20133MilanItaly; ^5^ Key Laboratory of Flexible Electronics (KLOFE) and Institute of Advanced Materials (IAM)Jiangsu National Synergetic Innovation Center for Advanced Materials (SICAM)Nanjing Tech University (NanjingTech)30 South Puzhu RoadNanjing211816China

**Keywords:** cathode interlayer, conjugated polyelectrolytes, inverted solar cells, light soaking effect, star‐shaped molecules

## Abstract

Three kinds of charged star‐shaped conjugated macroelectrolytes, named as PhNBr, TPANBr, and TrNBr, are synthesized as electron‐collecting interlayers for inverted polymer solar cells (i‐PSCs). Based on these well‐defined structured interlayer materials, the light soaking (LS) effect observed in i‐PSCs was studied systematically and accurately. The general character of the LS effect is further verified by studying additional i‐PSC devices functionalized with other common interlayers. The key‐role of UV photons was confirmed by electrochemical impedance spectroscopy and electron‐only devices. In addition, the ultraviolet photoelectron spectroscopy measurements indicate that the work function of the indium tin oxide (ITO)/interlayer cathode is significantly reduced after UV treatment. In these i‐PSC devices the LS effect originates from the adsorbed oxygen on the ITO substrates when oxygen plasma is used; however, even a small amount of oxygen from the ambient is also enough for triggering the LS effect, albeit with a weaker intensity. Our results suggest that the effect of adsorbed oxygen on ITO needs to be considered with attention while preparing i‐PSCs. This is an important finding that can aid the large‐scale manufacturing of organic solar cells via printing technologies, which do not always ensure the full protection of the device electrode substrates from oxygen.

## 1 Introduction

Organic photovoltaic technology is a promising method to utilize solar energy because of its capability of large‐scale and low‐cost production, lightweight physical characteristics, and the lucrative possibility of direct integration into flexible devices.^1^, ^2^, ^3^, ^4^ The inverted device architecture of polymer solar cells (i‐PSCs), where a bulk heterojunction (BHJ) photoactive layer is sandwiched between a transparent substrate as the bottom electron‐collecting (EC) electrode and an air‐stable high work function (WF) metal (Ag or Au) as the top hole‐collecting (HC) electrode, is an attractive approach for its superior long‐term stability compared to the initially implemented conventional device structures.^5^, ^6^ In i‐PSCs devices, however, the commonly utilized indium tin oxide (ITO) is not an ideal EC electrode for the typical polymer: fullerene BHJ active layer, because its high WF (≈4.7 eV) does not facilitate the formation of an Ohmic contact with the lowest unoccupied molecular orbital (LUMO) of the fullerene derivatives. Consequently, various kinds of interfacial materials including n‐type metal oxides (MOs) (ZnO, TiO*_x_*, MoO_3_–Al composite, and SnO*_x_*),^7^, ^8^, ^9^ alkali‐metal compounds,^10^ and cross‐linked fullerenes were explored to facilitate charge extraction.^11^

Apart from using n‐type MOs for bridging the WF_ITO_‐LUMO_fullerene_ energy gap, the use of EC interfacial materials such as conjugated polyelectrolytes (CPE) and nonconjugated polyelectrolytes (NCPE) was shown to be a smart way for tuning the WF_ITO_. By anchoring the EC interlayer material on the ITO surface, an electric dipole is formed that imposes a drastic effect by lowering the WF_ITO_.^12^ Polyfluorene derivatives with various kinds of polar pendant groups,^12^, ^13^, ^14^, ^15^, ^16^ poly(ethyleneimine),^17^ and ethoxylated poly(ethyleneimine)^18^ have been directly deposited on the surface of ITO in i‐PSCs, which show excellent capability in modifying the WF_ITO_ and improving device performance. Unlike n‐type MOs that commonly require a high annealing temperature (over 200 °C), organic‐based EC interlayer materials exhibit good solution processability at a low temperature and thus become more applicable for realistic applications of direct printing techniques on flexible plastic substrates.^19^, ^20^

In our previous work, star‐shaped conjugated macroelectrolyte EC materials have been developed.^21^, ^22^ This class of materials with a well‐defined chemical structure character could effectively circumvent the intractable problems accompanying the polymer counterparts in terms of molecular weight and polydispersity, catalyst residues, and poor reproducibility; their 3D molecular topology is also beneficial for improving the orthogonal solvent processability;^21^, ^22^, ^23^, ^24^, ^25^ it therefore appears as a promising portfolio of interfacial materials. In view of these advantages, the use of well‐defined material systems as EC interlayers is imperative for gaining further insight into the parameters that dictate the efficient charge extraction in i‐PSCs.

A common phenomenon in typical i‐PSCs incorporating MOs buffer layers (e.g., TiO*_x_* and ZnO), is the so‐called “light‐soaking (LS)” effect, which corresponds to an improvement of the device characteristics after the exposure of the device to light, especially to high‐energy UV photons.^26^, ^27^, ^28^, ^29^ Similar results have been documented when CPE‐based EC interlayers poly[(9,9‐bis(3′‐(*N,N*‐dimethylamino)propyl)‐2,7‐fluorene)‐alt‐2,7‐(9,9‐dioctylfluorene)] (PFN) and poly[3‐(6‐(*N*‐methylimidazolium)hexyl)‐2,5‐thiophene]bromide (P3lmHT) were used.^30^, ^31^ Nam suggested that the LS effect probably originates from the oxygen that is adsorbed during the processing of the substrates with oxygen plasma, which negatively impacts electron collection.^31^ However, a clear picture on the effects of oxygen adsorption on the resistivity, the energetic level alignment, and on the electron extraction ability of the ITO/CPE interface, as well as and on the role of UV light soaking in the improvement of the device performance is still lacking. Moreover, the reported LS effect observed in CPE‐functionalized i‐PSCs is still limited to the cases of PFN and P3ImHT EC interlayers and its generic role in i‐PSCs structures it is not yet confirmed. For these reasons, a systematic study on the LS effect observed in i‐PSC is highly required.

Herein, three kinds of charged star‐shaped molecules are utilized and investigated as EC interlayers for i‐PSCs. Based on these well‐defined structured interfacial materials, we provide a full map of the LS effects observed in organic EC‐interlayer‐based i‐PSCs. Although no MO interlayers were employed in the fabrication protocol of these studied i‐PSC devices, we found that all the devices exhibited LS effect. Detailed investigation on the variations of interfacial properties of the interlayer deposited onto ITO, before and after light irradiation, was performed by the ultraviolet photoelectron spectroscopy (UPS) and electrochemical impedance spectroscopy (EIS) to identify the principal role of UV photons in improving the device performance. In addition, we verified that the LS effects are observable in other MO‐free i‐PSC devices that utilized organic EC interlayers based on either a CPE poly[9,9‐bis(4′‐(6′′‐(*N,N,N*‐trimethylammonium)hexyloxy)phenyl)fluorene]‐bromide (PPFNBr) or a star‐shaped molecule with neutral dienthanolamino‐polar groups (TrOH).

## 2 Results and Discussion

**Scheme 1:**
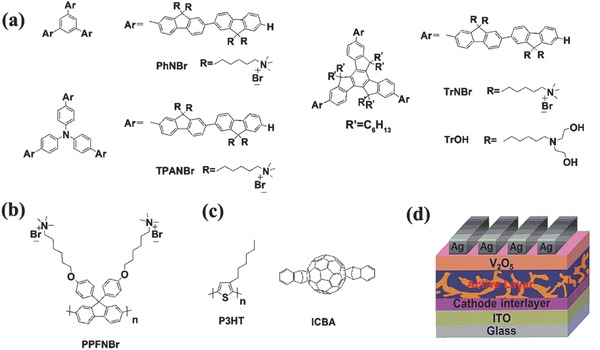
a) Chemical structures of the star‐shaped conjugated electrolyte interlayers (PhNBr, TPANBr, and TrNBr) and a neutral interlayer (TrOH) used in solar cell fabrication, b) chemical structure of one CPE‐based interlayer (PPFNBr) studied here, c) the active layer components employed: electron donor (P3HT) and acceptor (ICBA) materials, and d) the inverted device architecture.

The charged star‐shaped molecules used and the device structure studied are depicted in **Scheme** **1**. The star‐shaped molecules named PhNBr, TPANBr, and TrNBr, are composed of different hydrophobic cores from which three charged conjugated arms emanate. All devices in this study were prepared by including an oxygen plasma processing step of the ITO substrate for 10 min and by encapsulating the devices with glass and epoxy in an N_2_‐filled glovebox. A blend of poly(3‐hexylthiophene) (P3HT) as the electron‐donor and indene‐C_60_ bisadduct (ICBA) as the electron‐acceptor was used to form a BHJ active layer for the studied interlayer‐containing i‐PSCs. The high energy value of the LUMO level of ICBA is beneficial for achieving a large *V*_OC_ and for ensuring a reasonable device performance after incorporating P3HT as donor.^32^

The synthetic protocols followed for the preparation of the EC interlayers studied here are provided in the Supporting Information. We firstly investigated the interface properties of these EC interlayers. The sessile drop method was followed for determining the water contact angle (*θ*) on ITO substrates coated with PhNBr, TPANBr, and TrNBr, as deposited under the same spin‐coating condition (0.25 mg mL^−1^ in methanol, 5000 rpm) to form nearly 2 nm thick films, as shown in Figure S2 in the Supporting Information. The determined average contact angles of PhNBr, TPANBr, and TrNBr films were found to be 20.9°, 21.5°, and 22.3°, respectively, indicating similar hydrophilic properties of these substrates. Atomic force microscope imaging was performed to track the surface morphologies of the interlayer coated ITO substrates, and the corresponding images are presented in Figure S3 in the Supporting Information. No significant differences were found in the surface topography of the interlayer/ITO systems. Since the thickness of the utilized interlayers for device fabrication was very thin (nearly 2 nm), the roughness and topography of the films predominantly correspond to the surface properties of the ITO substrates used, indicating the presence of surface gaps in these interlayers.

**Figure 1:**
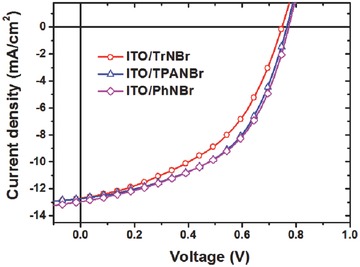
*J–V* characteristics under AM 1.5G irradiation at 100 mW cm^−2^ of these inverted devices with optimized TrNBr, TPANBr, and PhNBr interlayers.

Then, we fabricated i‐PSC devices with the general device geometry of ITO/EC interlayer/P3HT:ICBA/V_2_O_5_/Ag structure. *J–V* characteristics of the inverted devices with optimized interlayer thicknesses (nearly ≈2 nm, 0.25 mg mL^−1^) under AM 1.5G irradiation (100 mW cm^−2^) are presented in **Figure** **1**. In order to identify the optimized interlayer thickness, various interlayer spin‐casting conditions were tested where the interlayer thickness was determined by UV–vis absorption spectrum according to our previous report.^22^ For comparison, conventional devices with configuration of (ITO/poly(3,4‐ethylenedioxythiophene:poly(styrenesulfonate))(PEDOT:PSS)/P3HT:ICBA/Ca/Al) were prepared and the results are summarized in **Table** **1**. All the i‐PSCs devices exhibited outstanding stability for the period of 140 d as shown in Figure S5 in the Supporting Information. In order to ensure the validity of the comparison between the *J–V* characteristics of different devices shown in Figure 1, all cells were first illuminated by simulated solar light until reaching their optimum device performance. Devices based either on bare ITO substrates or on ITO substrates rinsed with methanol even after a long time of irradiation did not work. In contrast to the Ca/Al reference devices, all inverted devices exhibit significantly increased *J*_SC_, giving rise to improved PCE values. The performance of PhNBr‐based devices is very similar to that of TPANBr‐based devices, and their performance is a little better than the devices functionalized with TrNBr. In particular, the PhNBr‐based devices yielded the highest PCE of 5.00%, an ≈27% improvement compared with the Ca/Al reference device (PCE = 3.94%).^22^ Since the TrNBr, PhNBr, and TPANBr systems show similar interfacial properties (e.g., surface morphology, interfacial energy, and thickness), the apparent distinction between TrNBr and the other two material systems (PhNBr and TPANBr) can be explained based on the higher ion density (more hydrophilic part) of PhNBr and TPANBr than TrNBr, which is probably capable of providing more electron injection channels than that of TrNBr.^18^, ^33^

**Table 1:** Performance parameters of Ca/Al devices and inverted solar cells with various interlayers and spin‐casting conditions. The rate for spin‐casting was kept at 5000 rpm

Cathode	*V*_OC_ [V]	*J*_SC_ [mA cm^−2^]	FF [%]	PCEb) [%]	Averaged PCEc) [%]
Conventional Ca/Ala)	0.830	7.88	60.3	3.94	3.84 ± 0.12
ITO/TrNBr (0.13 mg mL^−1^)	0.730	11.70	44.9	3.84	3.74 ± 0.09
ITO/TrNBr (0.25 mg mL^−1^)	0.748	12.72	46.0	4.38	4.32 ± 0.04
ITO/TrNBr (0.50 mg mL^−1^)	0.754	11.08	49.9	4.17	4.09 ± 0.07
ITO/TPANBr (0.13 mg mL^−1^)	0.762	11.91	46.1	4.19	4.04 ± 0.14
ITO/TPANBr (0.25 mg mL^−1^)	0.766	12.75	50.6	4.94	4.74 ± 0.15
ITO/TPANBr (0.50 mg mL^−1^)	0.767	12.29	50.3	4.74	4.60 ± 0.10
ITO/PhNBr (0.13 mg mL^−1^)	0.747	12.65	45.5	4.30	4.19 ± 0.08
ITO/PhNBr (0.25 mg mL^−1^)	0.772	12.97	50.0	5.00	4.91 ± 0.08
ITO/PhNBr (0.50 mg mL^−1^)	0.800	11.87	48.5	4.60	4.44 ± 0.10

^a)^From our previous work^22^;

^b)^The device with best performance;

^c)^Eight devices measured in all. Note that all the parameters for i‐PSCs were recorded after light illumination until saturated PCE values.

**Figure 2:**
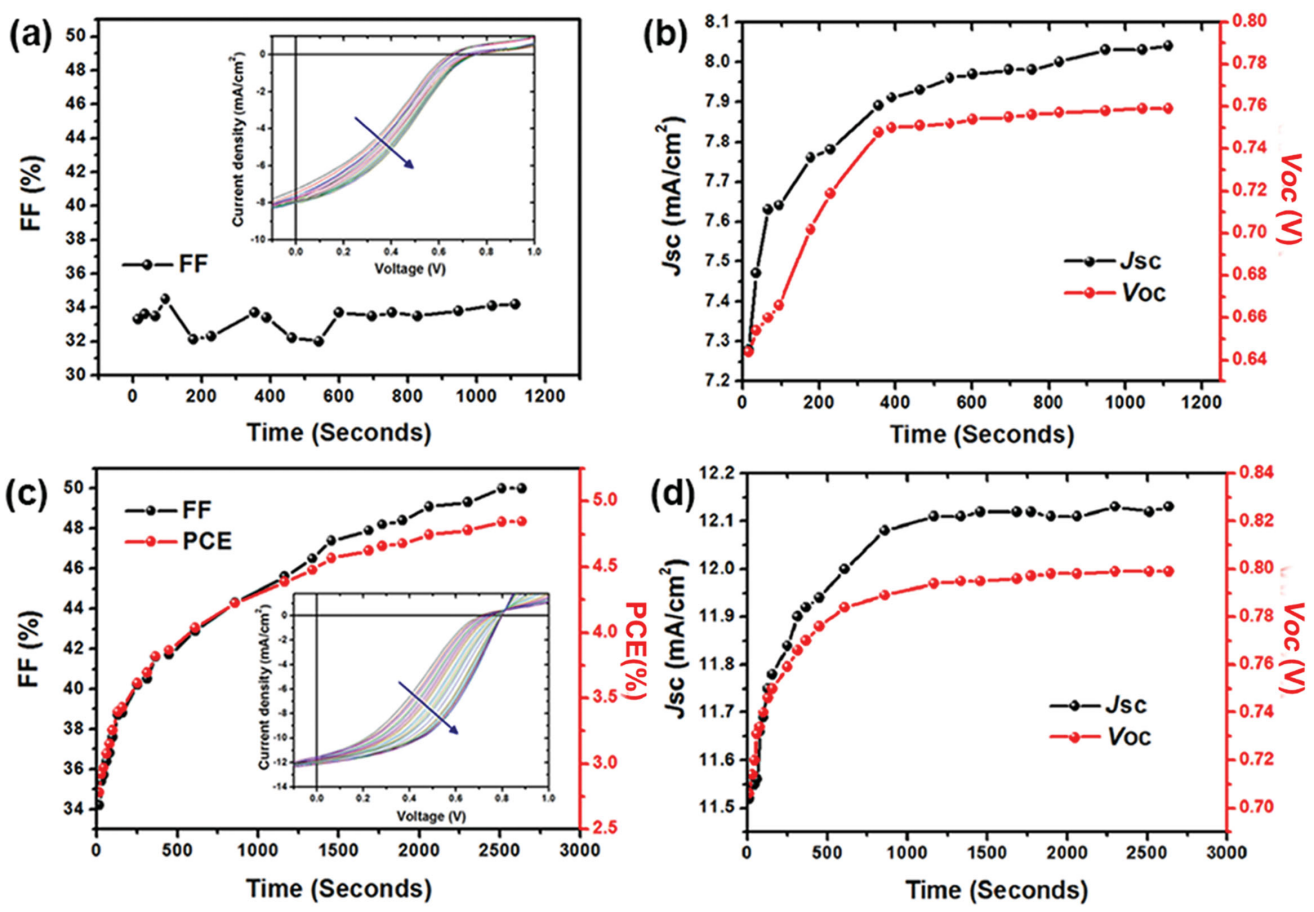
PhNBr‐based i‐PSCs devices: the variation of a,c) FF, PCE, *J–V* characteristics (inset), and b,d) *J*_SC_ and *V*_OC_ with increasing light illumination time (AM 1.5G, 100 mW cm^−2^); a,b) measured with a 405 nm UV blocking filter; c,d) measured without the UV blocking filter.

Owing to its best performance among the studied star‐shaped interlayer materials, we chose PhNBr as the model compound to unravel the origins of the LS effect. PhNBr‐containing i‐PSCs devices were fabricated. For a fresh device, an fill factor (FF) = 34.2% and a power conversion efficiency (PCE) = 2.5% were found corresponding to an S‐shaped *J–V* curve. However, after nearly 2600 s of illumination under simulated sunlight (AM 1.5G, 100 mW cm^−2^), the inflection point in the *J–V* curve disappeared and an overall improvement in the device parameters was achieved with a significant increase in *J*_SC_, *V*_OC_, FF, and PCE rising from 11.5 to 12.15 mA cm^−2^, 0.70 to 0.80 V, 34% to 50%, and 2.75% to 4.80%, respectively (see **Figure** **2**c,d). In order to identify the effect of UV light on the performance of the devices, we have repeated this measurement by filtering out the UV component of the simulated solar light with the use of a UV blocking filter (405 nm). Although a continuous increase was found for the *V*_OC_ and *J*_SC_ device parameters, no effect was seen on the FF parameter that did not exhibit substantial differences before and after prolonged illumination (Figure 2a,b). The obtained results are in line with previous suggestions that UV light illumination plays a major role in the recovery of the FF in the devices.^31^ Additional experiments were performed where only UV light (365 nm) was used during the prolonged device illumination (Figure S6, Supporting Information). The overall enhanced parameters (*J*_SC_, *V*_OC_, and FF) and corresponding PCE verified the key role of UV photons.

In order to demonstrate the general character of the UV‐light induced LS effect we studied i‐PSCs functionalized with conjugated‐electrolyte and neutral organic EC interlayers. First, we tested the more commonly utilized CPE EC interlayer of PPFNBr in i‐PSC devices with a photoactive layer of P3HT:PC71BM. The material structures and *J–V* characteristics of the tested devices are shown in Figure S7 in the Supporting Information, where the enhancement in FF and PCE can be seen after light irradiation. Moreover, i‐PSC devices with P3HT:ICBA as an active layer functionalized with a neutral star‐shaped interlayer bearing the diethanolamino‐moiety (TrOH) also exhibited the LS effect as shown in Figure S8 in the Supporting Information. Finally, we further tested PhNBr‐coated ITO electrodes in nonfullerene‐based i‐PSC devices with an active layer comprising a blend of poly[4,8‐bisbenzo[1,2‐b:4,5‐b′]dithiophene‐2,6‐diyl‐alt‐4‐thieno[3,4b]‐thiophene‐2,6‐diyl] (PBDTTT‐E‐O) and the *N*,*N*′‐bis(1‐ethylpropyl)‐perylene‐3,4,9,10‐tetracarboxylic diimide (EP‐PDI) (Scheme S1, Supporting Information). Similar to the P3HT:ICBA‐based i‐OSCs, these devices also exhibit LS issues. The expected performance could only be achieved after light irradiation, with improved PCE from 0.51% to 1.36%, as shown in Figure S9 in the Supporting Information and summarized in Table S1 in the Supporting Information.

One explanation suggested by a previous literature is that the adsorbed oxygen can probably lead to the degradation of organic semiconductors.^26^, ^34^ Interestingly, in our study the LS effect was found to be reversible; the LS‐induced saturated *J–V* device characteristics tended to return to the initially obtained S‐shaped *J–V* curve, after storing the devices in ambient condition in dark for 30 d (Figure S10, Supporting Information). The possible changes to the chemical structure of the interlayer materials after prolonged UV irradiation should not be held responsible for the LS effect. Instead, prolonged UV light illumination of the devices leads to temporary photoinduced changes. This phenomenon is reverse to previous reported *J–V* reanimation in aging ITO/PEDOT:PSS electrode after keeping in dark for a duration.^35^

**Figure 3:**
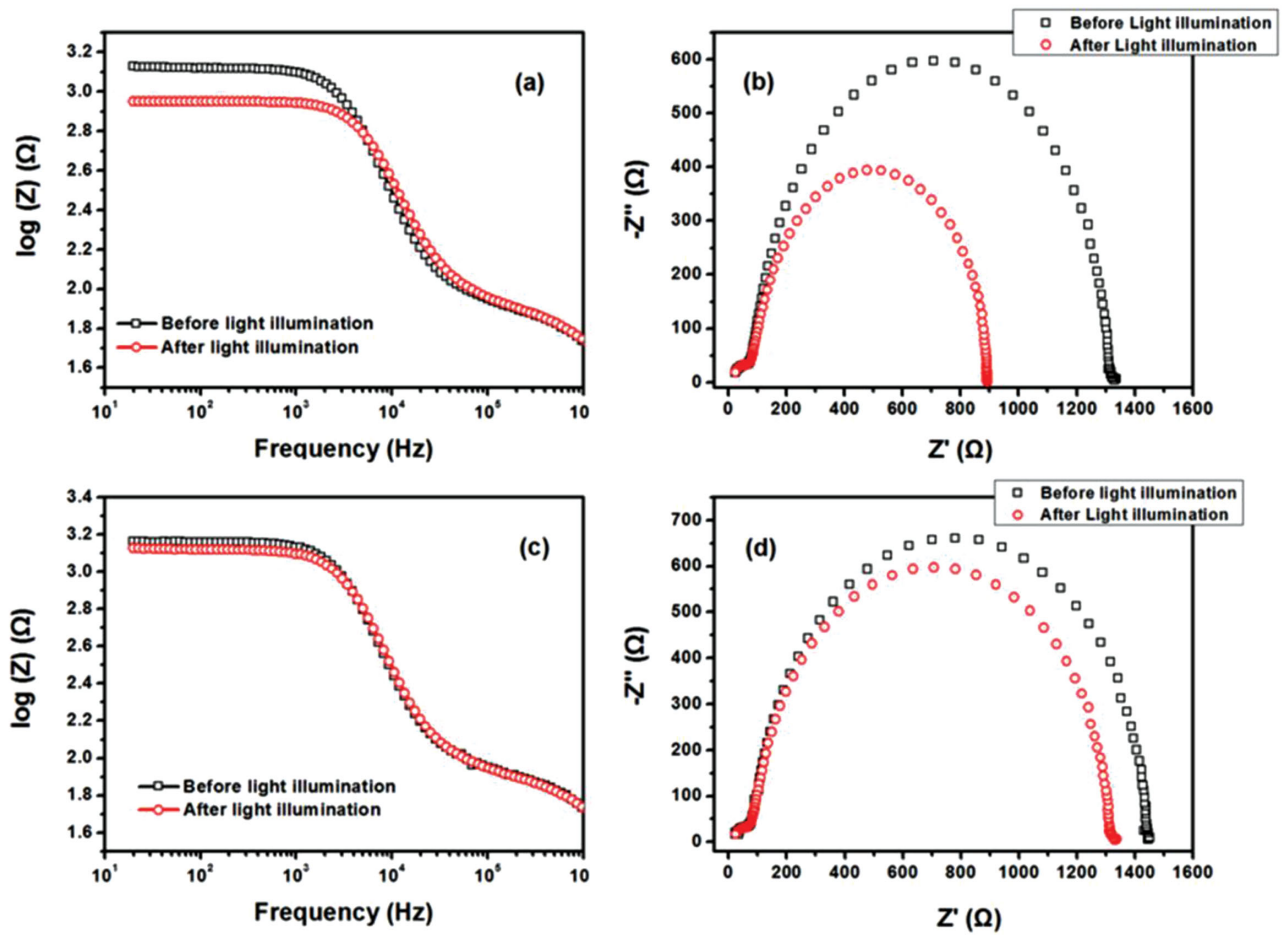
a,c) The Bode plot, and b,d) Nyquist plot of inverted solar cells with PhNBr interlayer measured before (black squares) and after light irradiation (red circles) until the saturated PCE value. In panels (a) and (b), the device was measured under irradiation at AM 1.5G 100 mW cm^−2^, and in panels (c) and (d), one UV blocking filter (405 nm) was utilized to cut UV light. All the measurements were performed at *V*_OC_. It was noted that both of them were not fresh devices, which had been illuminated to saturated PCE value and then be kept in dark for 30 d before ESI measurements. During this period, the devices were kept in a digital dry box with humidity of 25% at 20 °C.

Once we have established the occurrence of the UV‐light‐induced LS effect in several i‐PSC devices equipped with different types of EC interlayers, we explore its consequences in electrical conductivity and charge extraction capabilities of the i‐PSC devices. The technique of EIS has been widely applied to analyze the electrical properties of interfaces.^36^ We have performed EIS characterization on the PhNBr‐based i‐PSC devices when operating the cells under *V*_OC_ during light illumination (with and without UV light). Since the *V*_OC_ parameter increases rapidly during the first minutes of continuous illumination, the EIS characterization of a fresh device was not possible. Thus, we turned to the utilization of devices which had already experienced a cycle of continuous illumination up to the saturation limit and then kept in ambient for a month until restoring their initial kink shaped *J–V* characteristics (Figure S9, Supporting Information). According to the corresponding EIS data (**Figure** **3**), the devices exhibit significantly reduced bulk resistance after prolonged irradiation with simulated sunlight, as indicated by the decreased radius seen in the Nyquist plot of Figure 3. This observation suggests that light irradiation improves conductivity. Additional EIS measurements, after filtering out the UV component of the utilized light, found that the bulk resistance is not reduced as much as when the UV light is on.

The observed increase in bulk conductivity when the PhNBr interlayer is used, may be attributed to increased charge transport and/or to improved charge extraction. In order to disentangle the two effects we have tested electron‐only P3HT:ICBA devices with PhNBr interfacial layer. For the device configuration of ITO/PhNBr/P3HT:ICBA/Ca/Al, we found an improvement in the *J–V* device characteristics, after exposure of the device to simulated sunlight for 10 min (see Figure S11 in the Supporting Information). Following prolonged light exposure, the *J–V* characteristics of the ITO/PhNBr‐based devices become more symmetric, suggesting that light‐soaking lowers the energetic barrier for electron extraction.

**Figure 4:**
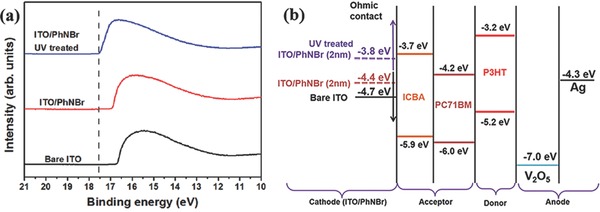
a) UV photoelectron spectroscopy for bare oxidized ITO, ITO/PhNBr (2 nm), and ITO/PhNBr (2 nm) after UV treatment (375 nm, 10 mW cm^−2^). b) Energy level diagrams of the inverted device components under the flat band condition.

Zhou et al. proposed that the adsorbed oxygen can strongly influence the WF of ITO, and the oxygen can be deposited after UV irradiation, resulting in lower WF of ITO,^37^ which is in line with the LS effect of i‐PSCs with ITO/EC interlayer electrodes. We experimentally confirm that the origin of the LS effect of ITO/EC interlayer comes from the variation of the Fermi levels due to the presence of dipoles formed after O_2_ adsorption which have an opposite direction to the dipole induced by the EC interlayer. We have performed UPS for PhNBr/ITO electrodes and the effect of UV irradiation on the electronic properties of these systems was assessed. The ITO substrates were processed in identical fashion to those used for device fabrication. As presented in **Figure** **4**a, the shift of the secondary electron cutoff (*E*_SE_) after 2 nm thick PhNBr interlayer without prolonged light exposing indicates a vacuum level (VL) shift of 0.3 eV. However, following exposure to UV light (375 nm, 10 mW cm^−2^) for 30 min, the use of a 2 nm thick PhNBr interlayer is accompanied by a large VL shift of 0.9 eV. Therefore a rearrangement of energy levels occurs at the ITO/PhNBr interface after UV irradiation. Based on the UPS results, the corresponding energy diagram shown in Figure 4b can be suggested. It is found that the main reason for the improved device performance after UV treatment is the optimal matching of the WF of the bottom electrode with the LUMO level of the herein utilized electron acceptors (ICBA, PC70BM).

**Figure 5:**
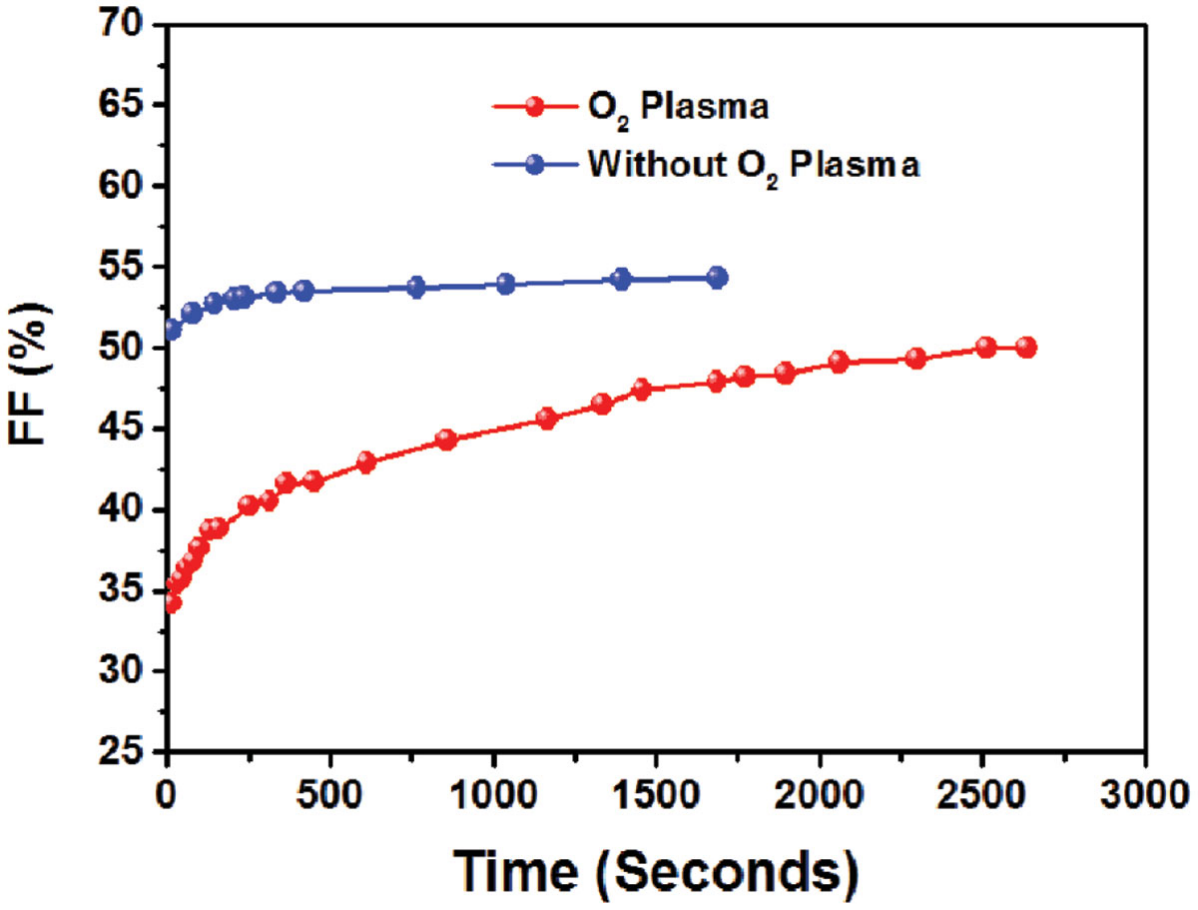
Time‐dependent variation of FF of PhNBr‐based inverted devices with and without oxygen plasma processing.

The intensity of the LS effect is expected to be proportional to the initial amount of adsorbed oxygen onto the ITO electrode. We performed a comparative measurement for two sets of PhNBr‐based i‐PSC devices of ITO/PhNBr/P3HT:ICBA/V_2_O_5_/Ag; one set processed with and the other without oxygen plasma treatment. For the latter set, the ITO substrates were stored in N_2_‐filled glovebox for 3 d before device fabrication. The devices that adsorbed less oxygen exhibited a negligible albeit observable LS effect in the corresponding *J–V* characteristics. In contrast, for the devices treated with oxygen plasma, a recovery in the FF parameter was seen after directly irradiating the device under simulated solar light. **Figure** **5** shows the temporal evolution of the FF and the percentage of initial FF values as obtained after the continuous illumination of the devices by a solar simulator with an output intensity of 1 Sun (AM 1.5G). All the data were recorded until reaching their saturated PCE values. A 16% improvement (from 34% to 50%) was found in the FF parameter for the devices treated with O_2_ plasma. Notably, the devices processed without O_2_ plasma exhibited a high FF initially (51%), and after 1600 s of illumination, only a slightly enhanced FF value to 54% was found. Moreover, for these devices, the *V*_OC_ and *J*_SC_ parameters were found to be low but after prolonged illumination saturation was reached with *V*_OC_ being increased by 15% (Figure S12, Supporting Information). The observed weak LS effect and the initially low *V*_OC_ in the devices not processed by O_2_ plasma verify the essential role of oxygen adsorption during O_2_ plasma treatment. Moreover the observed LS effect, albeit weak, in these well‐encapsulated devices suggests that even a minute amount of oxygen from the ambient can be adsorbed on ITO during the preliminary processing steps prior encapsulation and can influence the energy level alignment of ITO/EC interlayer interface.

## 3 Conclusions

In conclusion, we have successfully fabricated i‐PSCs using the PhNBr, TPANBr, and TrNBr star‐shaped conjugated macroelectrolytes as EC interlayers on the top of ITO electrode substrates. Based on these well‐defined structured interlayer materials, the LS effect observed in i‐PSCs was studied systematically and accurately. The general character of the LS effect was demonstrated by studying additional i‐PSC devices functionalized with the more commonly utilized CPE of PPFNBr interlayer and with the neutral star‐shaped interfacial material TrOH. The key role of UV photons was confirmed by EIS and electron‐only devices. In addition, UPS measurements indicated that the WF of the ITO/interlayer cathode is significantly reduced after UV treatment. In these i‐PSC devices the LS effect originates from the adsorbed oxygen on the ITO substrates when oxygen plasma is used; however, even a small amount of oxygen from the ambient is also enough for triggering the LS effect, albeit with a weaker intensity. Our results suggest that the effect of adsorbed oxygen on ITO need to be considered with attention while preparing i‐PSCs. This is a meaningful finding that can aid the large‐scale manufacturing of organic solar cells via printing technologies, which do not always ensure the full protection of the device electrode substrates from oxygen.

## Supporting information

As a service to our authors and readers, this journal provides supporting information supplied by the authors. Such materials are peer reviewed and may be re‐organized for online delivery, but are not copy‐edited or typeset. Technical support issues arising from supporting information (other than missing files) should be addressed to the authors.

Supplementary
