# Supplementary material for: Understanding the Light Soaking Effects in Inverted Organic Solar Cells Functionalized with Conjugated Macroelectrolyte Electron‐Collecting Interlayers
Source: Adv Sci (Weinh). 2015 Dec 16;3(2):1500245. doi: 10.1002/advs.201500245 (PMC5115465; doi:10.1002/advs.201500245)
Supplement: Supplementary file 1 — Supplementary [file ADVS-3-0p-s001.pdf]

Copyright WILEY-VCH Verlag GmbH & Co. KGaA, 69469 Weinheim, Germany, 2015.

ADVANCED SCIENCE
Open Access

Supporting Information

for *Adv. Sci.*, DOI: 10.1002/advs. 201500245

Understanding the Light Soaking Effects in Inverted Organic Solar Cells Functionalized with Conjugated Macroelectrolyte Electron-Collecting Interlayers

Weidong Xu , Ruidong Xia , Tengling Ye , Li Zhao , Zhipeng Kan , Yang Mei , Congfei Yan , Xin-Wen Zhang , Wen-Yong Lai , Panagiotis E. Keivanidis ,* and Wei Huang**

Copyright WILEY-VCH Verlag GmbH & Co. KGaA, 69469 Weinheim, Germany, 2013.

Supporting Information for

Understanding the Light Soaking Effects in Inverted Organic Solar Cells Functionalized with Conjugated Macroelectrolyte Electron-Collecting Interlayers

Weidong Xu, Ruidong Xia, Tengling Ye, Li Zhao, Zhipeng Kan, Yang Mei, Congfei Yan, Xin-Wen Zhang, Wen-Yong Lai, Panagiotis E. Keivanidis* and Wei Huang**

W. Xu, Prof. Dr. R. Xia, L. Zhao, Y. Mei, C. Yan, Prof. Dr. W.-Y. Lai, Prof. Dr. W. Huang
Key Laboratory for Organic Electronics and Information Displays (KLOEID) & Institute of Advanced Materials (IAM), Jiangsu National Synergetic Innovation Center for Advanced Materials (SICAM), Nanjing University of Posts & Telecommunications, 9 Wenyuan Road, Nanjing 210023, China, E-mail: iamwylai@njupt.edu.cn

Assist. Prof. Dr. P. E. Keivanidis
Cyprus University of Technology, Department of Mechanical Engineering and Materials Science and Engineering, Dorothea Bldg 511, 45 Kitiou Kyprianou str., Limassol 3041, Cyprus, E-mail: p.keivanidis@cut.ac.cy

Dr. Z. Kan
Center for Nano Science and Technology@PoliMi
Istituto Italiano di Tecnologlia *via* G. Pascoli 70/3, I-20133 Milano, Italy

Assoc. Prof. Dr. T. Ye
Department of Chemistry, Harbin Institute of Technology, Harbin, 150001, China

Prof. Dr. W.-Y. Lai, Prof. Dr. W. Huang
Key Laboratory of Flexible Electronics (KLOFE) & Institute of Advanced Materials (IAM), Jiangsu National Synergetic Innovation Center for Advanced Materials (SICAM), Nanjing Tech University (NanjingTech), 30 South Puzhu Road, Nanjing 211816, China, E-mail: wei-huang@njtech.edu.cn

1. General Methods.

NMR spectra were recorded on a Bruker Ultra Shield Plus 400 MHz NMR (^{1}H: 400 MHz, ^{13}C: 100 MHz). The molecular weight of intermediates was measured by a Bruker matrix-assisted laser desorption/ionization time of flight mass spectrometry (MALDI-TOF MS) with CF_3COOAg and trans-2-[3-(4-tert-Butylphenyl)-2-metheyl-2-propenylidene] malononitrile (DCTB) as the matrix. UV-Vis spectra were measured with Shimadzu UV-Vis-NIR spectrophotometer was recorded with Shimadzu Luminescence Spectrometer LS 50. Atom Force Microscopy (AFM) measurements of surface morphology were conducted on the Bruker ScanAsyst AFM in auto scan (AC) mode. Film water contact angles were measured with a CAM 200 (KSV Instrument LID) and the photos were taken with a BASLER A602f-2 camera. ESI measurements were performed on an auto-lab system. UPS measurements was conducted in a Kratos AXIS Ultra DLD ultrahigh-vacuum (UHV) surface analysis system. UPS analysis with an unfiltered HeI (21.2 eV) gas discharge lamp was performed to characterize the valence states and the vacuum level (VL), showing a total instrumental energy resolution of 100 meV. All the samples were kept in a high vacuum chamber overnight before performing the measurements.

2. Materials and Synthesis

2.1 Materials

All reagents and solvents, unless otherwise specified, were obtained from Aldrich, Acros, and TCI Chemical Co. Meanwhile, they were used as received. All manipulations involving air sensitive reagents were performed under an atmosphere of dry argon. The synthesis routes towards PPFNBr is according to our previous work.[1-2] TrNBr and TrOH have been reported in our previous work.[3] The average molecular weight (M_n) estimated by gel permeation chromatography (GPC) using THF as the eluent was 24600 (with a polydispersity of 2.5) for the neutral precursor of PPFNBr. The active materials including P3HT, ICBA, PC70BM were

sourced from Solenne and used as received. PBDTTT-E-O and EP-PDI materials were purchashed from Solarmer Energy Inc. and used as received without further purification.

2.2 Synthesis

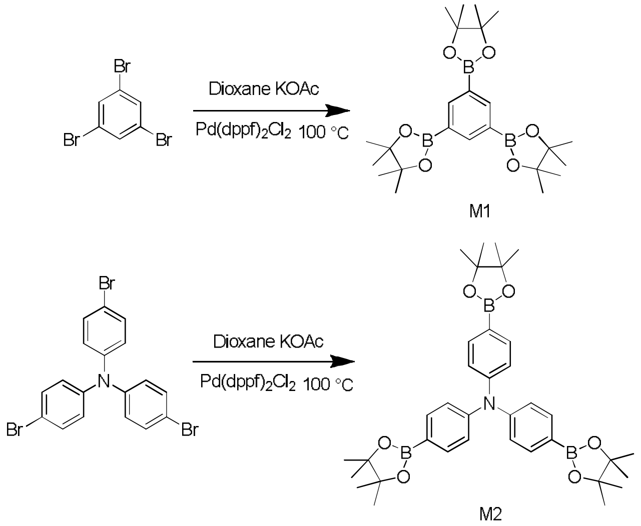

1,3,5-Tris(4,4,5,5-tetramethyl-1,3,2-dioxaborolan-2-yl)benzene (M1): A mixture of 1,3,5-tribromobenzene (1.57 g, 5 mmol), KOAc (2.94 g, 30 mmol), and bis(pinacolato)diboron (4.57 g, 18 mmol) in anhydrous dioxane (20 mL) was charged under nitrogen and stirred for 15 min, then $Pd(dppf)_2Cl_2$ was added under nitrogen. The mixture was stirred at 100°C for 24 h. After cooling to room temperature, the resulting mixture was diluted with water and extracted with CH_2Cl_2 (3 × 50 mL). The combined organic layer was washed with water (3 × 50 mL) and dried over anhydrous $MgSO_4$. The solvent was evaporated and the residue was purified by column chromatography (silica gel, 20% CH_2Cl_2 in hexane), then it was recrystallized from acetone to afford M1 as a white solid (1.95 g, 86%). ^{1}H NMR (400 MHz, $CDCl_3$): δ 8.36 (s, 3H), 1.33 (s, 36H).

tris(4-(4,4,5,5-tetramethyl-1,3,2-dioxaborolan-2-yl)phenyl)amine (M2): A flask charged with tris(4-bromophenyl)amine (0.568 g, 1 mmol), bis(pinacolato)diborane (0.38 g, 1.5 mmol), potassium acetate (0.37 g, 3.75 mmol), $Pd(dppf)_2Cl_2$ (25 mg, 0.03 mmol), and 10 mL of anhydrous dioxane was degassed for 15 min. After the mixture was stirred at 95°C for 12 h, it was cooled to room temperature and then poured into ice water (100 mL). The mixture was

then extracted with $CHCl_3$, and the combined organic layers were dried over anhydrous $MgSO_4$. After the solvent was evaporated, the residue was purified by chromatography using silica gel (hexane: $CHCl_3$ = 2:1) to afford crude products. At last, the crude **M2** was puried by recrystallization (465 mg, 74%) as a white solid. ^{1}H NMR (400 MHz, $CDCl_3$): δ 7.68 (d, J = 8.2 Hz, 6H), 7.07 (d, J = 8.2 Hz, 6H), 1.34 (s, 36H).^{13}C NMR (100 MHz, $CDCl_3$): δ 149.80, 135.93, 123.49, 83.68, 24.88, 1.03.

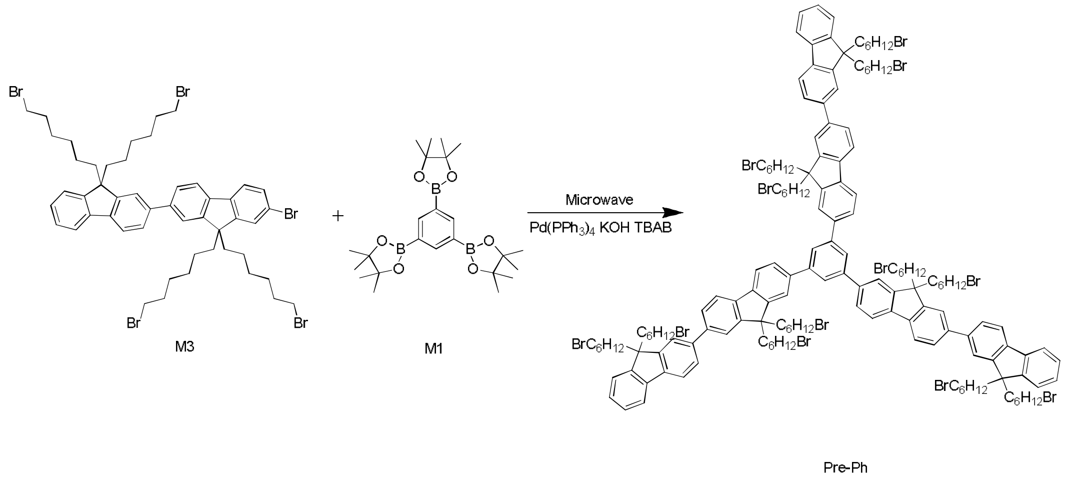

1,3,5-Tris(9,9,9',9'-tetrakis(6-bromohexyl)-9H,9'H-[2,2'-bifluoren]-7-yl)benzene (Pre-Ph): A flask charged with **M1** (618 mg, 1 mmol), 2,7-dibromo-9,9-bis (6′-bromohexyl) fluorene (**M3**) (1.30 g, 2 mmol), $Pd(PPh_3)_4$ (10 mg), tetrabutylammonium bromide (TBAB) and potassium carbonate (1.38 g, 10 mmol) in toluene/water (2:1, 15 mL) was degassed for 15 min. After being refluxed for 24 h, it was cooled to room temperature and then extracted with CH_2Cl_2 (25 mL× 3). The organic layer was washed with water, brine and dried with anhydrous $MgSO_4$. The solvent was removed and the residue was purified by silica gel column using hexane/CH_2Cl_2 (2:1) as the eluent to afford **Pre-Ph** (670 mg, 65%) as a white solid. ^{1}H NMR (400 MHz, $CDCl_3$): δ 7.95-7.64 (m, 33 H), 7.41-7.32 (m, 9 H), 3.29 (t, J = 6.8 Hz, 24 H), 2.11 (d, J = 32.0 Hz, 24 H), 1.73-1.64 (m, 24 H), 1.25-1.08 (m, 48 H), 0.78 (d, J = 37.3 Hz, 24 H). ^{13}C NMR (100 MHz, $CDCl_3$): δ 151.60, 151.47, 151.22, 150.64, 143.02, 140.81, 140.74, 140.57, 140.54, 140.43, 140.31, 140.01, 127.23, 127.04, 126.66, 126.46, 126.29, 125.35, 125.34, 122.90, 121.81, 121.36, 121.27, 120.30, 120.10, 119.90, 55.44, 55.17,

40.44, 40.32, 34.06, 34.03, 32.71, 32.69, 29.20, 29.13, 27.87, 27.83, 23.79, 23.67. MALDI-TOF MS (*m/z*): calcd for $C_{156}H_{186}Br_{12}$, Mol. Wt.: 3019.99; found: *m/z* 3019.38.

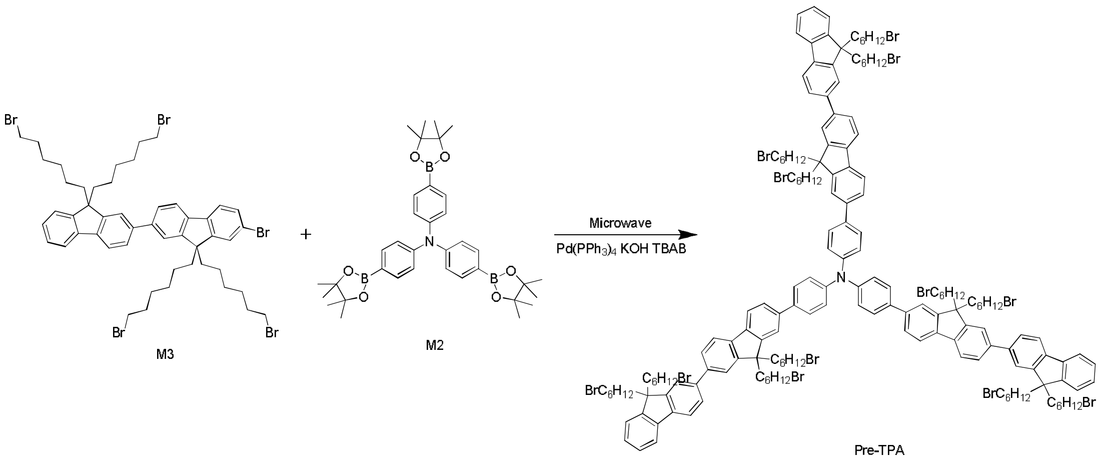

Tris(4-(9,9,9',9'-tetrakis(6-bromohexyl)-9H,9'H-[2,2'-bifluoren]-7-yl)phenyl)amine (Pre-TPA): A flask charged with **M2** (618 mg, 1 mmol), 2,7-dibromo-9,9-bis(6′-bromohexyl) fluorene (**M3**) (1.30 g, 2 mmol), $Pd(PPh_3)_4$ (10 mg), tetrabutylammonium bromide (TBAB) and potassium carbonate (1.38 g, 10 mmol) in toluene/water (2:1, 15 mL) was degassed for 15 min. After being refluxed for 24 h, it was cooled to room temperature and then extracted with CH_2Cl_2 (25 mL×3). The organic layer was washed with water, brine and dried with anhydrous $MgSO_4$. The solvent was removed and the residue was purified by silica gel column using hexane/CH_2Cl_2 (2:1) as eluent to afford **Pre-TPA** (670 mg, 65%) as a green solid. ^{1}H NMR (400 MHz, $CDCl_3$): δ 7.81 (t, J = 6.7 Hz, 9 H), 7.75 (d, J = 7.7 Hz, 3 H), 7.69-7.60 (m, 24 H), 7.38-7.32 (m, 15 H), 3.29 (t, J = 6.7 Hz, 24 H), 2.14-2.03 (m, 24 H), 1.72-1.64 (m, 24 H), 1.16-1.09 (m, 24 H), 0.87-0.69 (m, 48 H). ^{13}C NMR (100 MHz, $CDCl_3$): δ 193.40, 151.41, 151.39, 151.18, 150.61, 150.57, 146.79, 143.38, 140.79, 140.46, 140.12, 139.83, 139.54, 138.92, 136.03, 132.92, 130.51, 129.02, 127.98, 127.17, 127.00, 126.34, 126.22, 125.79, 124.53, 123.99, 122.87, 121.25, 121.21, 120.92, 120.18, 120.07, 120.05, 119.85, 118.45, 55.25, 55.12, 40.34, 40.26, 33.99, 33.96, 32.64, 32.63, 29.09, 29.07, 27.78, 27.76, 23.67, 23.61. MALDI-TOF MS (*m/z*): calcd for $C_{168}H_{195}Br_{12}N$, Mol. Wt.: 3187.20; found: *m/z* 3193.37.

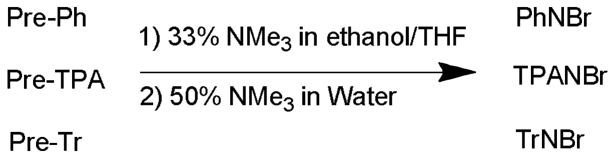

General methods to synthesize PhNBr and TPANBr: 150 mg of **Pre-Tr** was dissolved into 10 mL of THF, and 5 mL of trimethylamine solution in ethanol (33%) was then added. After stirred at room temperature for 48 h, it can be found some solid precipitated. Then another dose of 50% TMA aqueous solution was added until all the solids dissolved. After stirred for another 24 h, the solvent was evaporated at vacuum. After washed by THF for three times, it was collected by centrifugation and dried overnight in vacuum at 50℃ to afford **PhNBr** (165 mg, 92.3%) as a white solid. **PhNBr:** ^{1}H NMR (400 MHz, MeOD): δ 8.06-7.73 (m, 33 H), 7.41 (d, J = 37.9 Hz, 9 H), 3.24 (s, 24 H) 3.04 (s, 108 H), 2.25 (d, J = 56.4 Hz, 24 H), 1.68-1.52 (br, 24 H), 1.24 (d, J = 22.2 Hz, 48 H), 0.84-0.60 (br, 24 H). **TPANBr** (green solid): ^{1}H NMR (400 MHz, MeOD): δ 7.95-7.65 (d, J = 40.5 Hz, 36 H), 7.50-7.25 (br, 15 H), 3.25 (br, 24 H), 3.04 (s, 108 H), 2.32-2.10 (br, 24 H), 1.69-1.50 (br, 24 H), 1.30-1.10 (br, 48 H), 0.85-0.60 (br, 24 H).

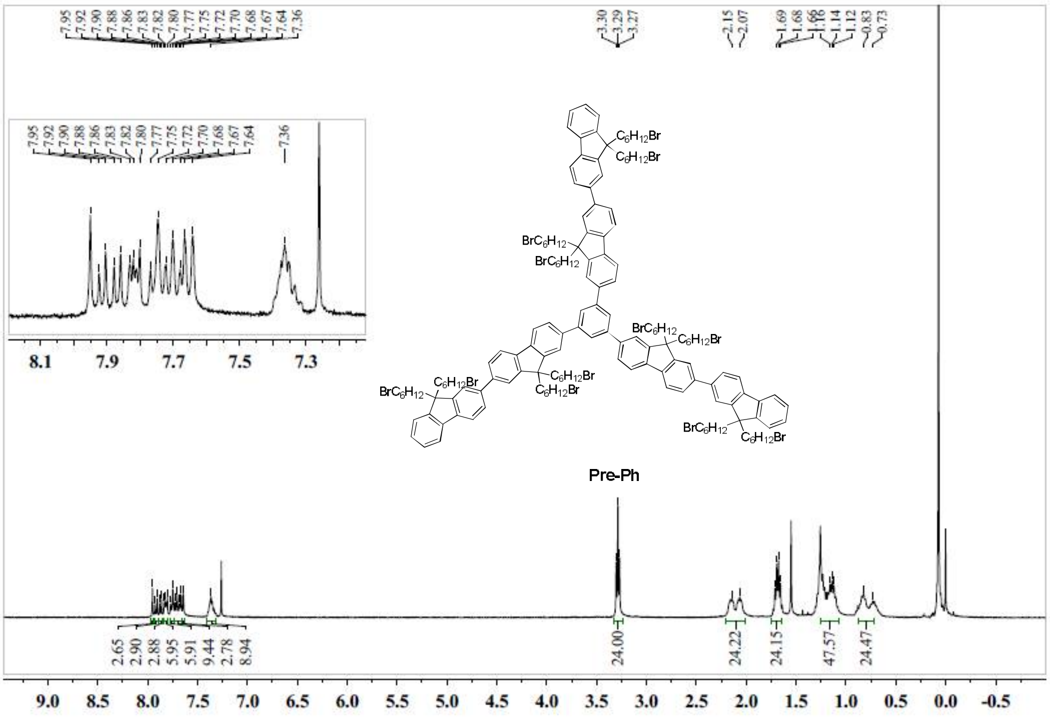

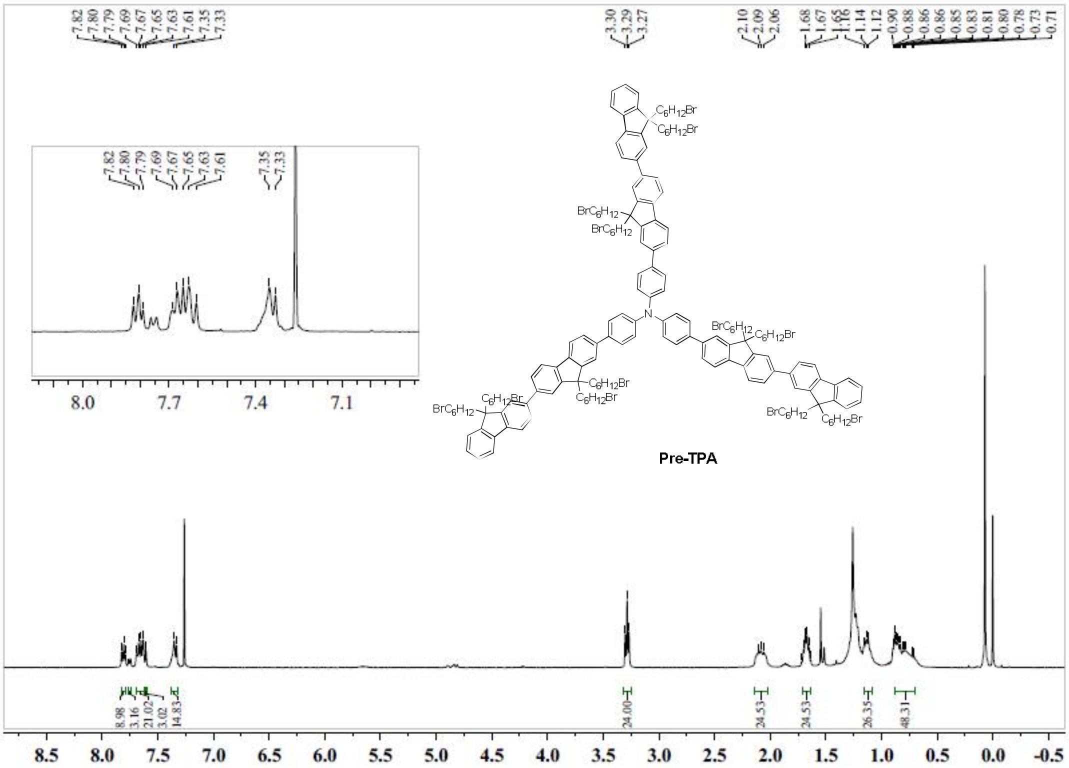
Pre-TPA
8.5 8.0 7.5 7.0 6.5 6.0 5.5 5.0 4.5 4.0 3.5 3.0 2.5 2.0 1.5 1.0 0.5 0.0 -0.5

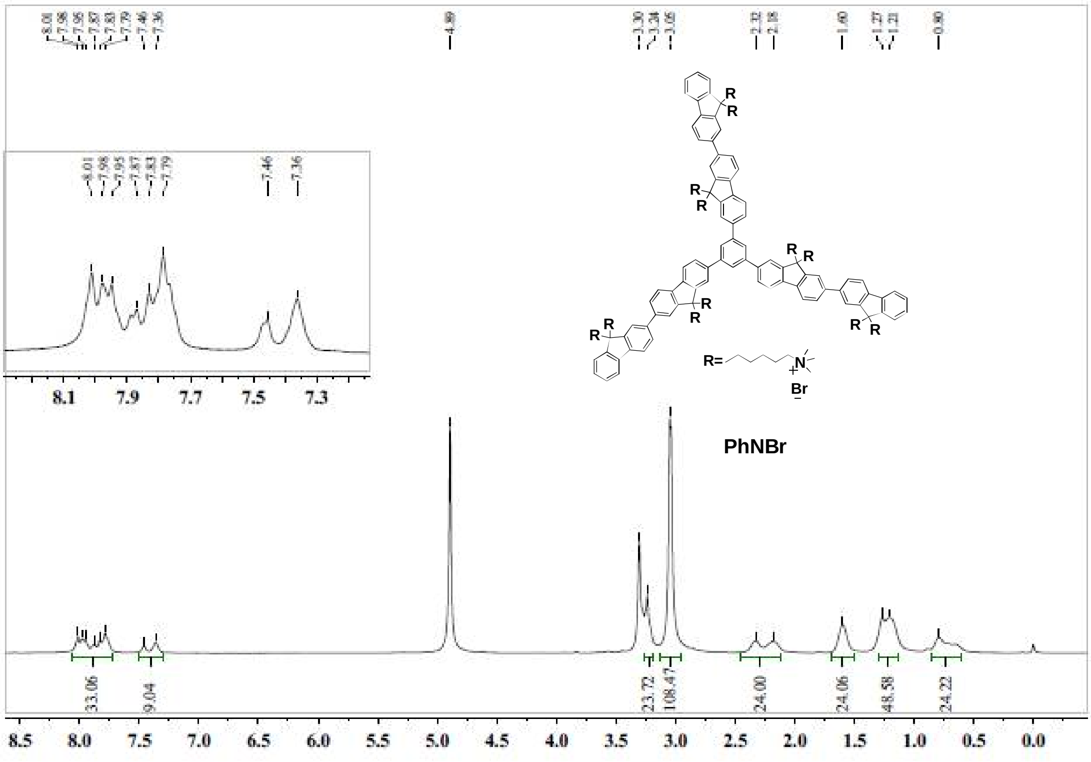
PhNBr
8.5 8.0 7.5 7.0 6.5 6.0 5.5 5.0 4.5 4.0 3.5 3.0 2.5 2.0 1.5 1.0 0.5 0.0

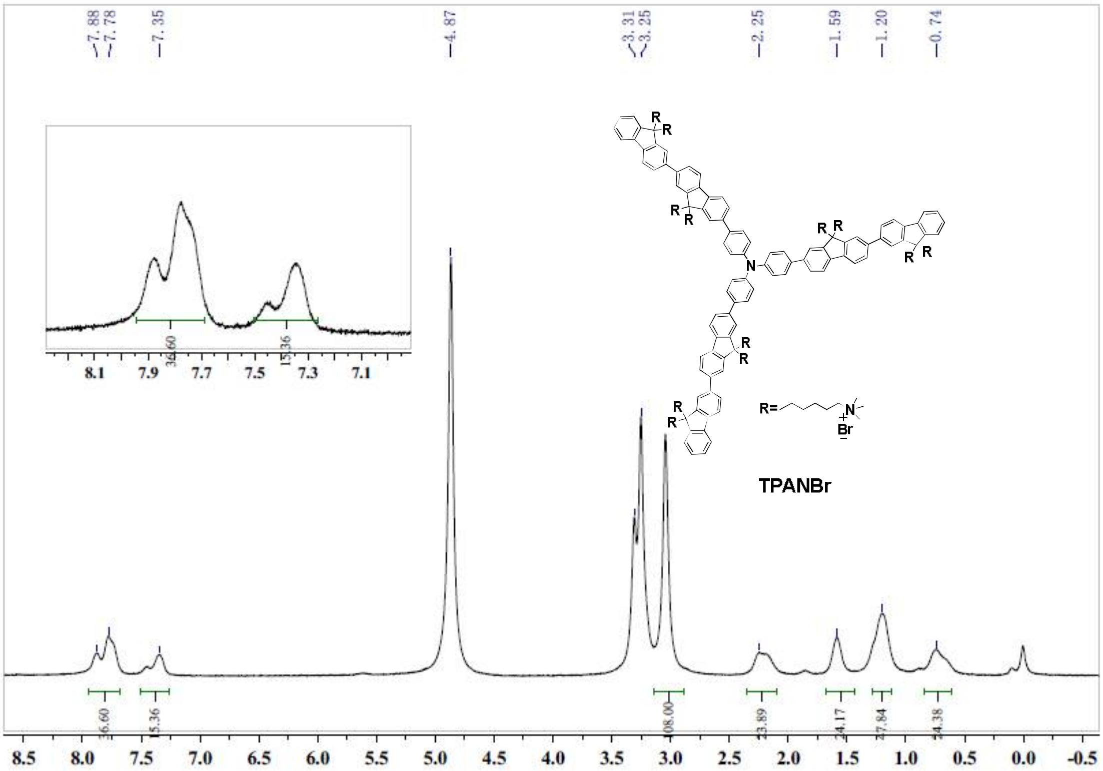

3. Device fabrication and characterization

The glass/ITO substrates were pre-cleaned with acetone, isopropanol, and a special detergent and dried under a flow of dry nitrogen. Before the deposition of the organic layers, the ITO substrates were cleaned in O_2 plasma for 10 min. Inverted PSCs were fabricated with structure ITO/interlayer/active layer/V_2O_5 or MoO_3/Ag and (non-inverted) reference PSCs with structure ITO/PEDOT:PSS/ P3HT:ICBA/Ca/Al. For the inverted PSCs, all the charged star-shaped conjugated macromolecules (including PhNBr, TPANBr, TrNBr) were spin-coated from their various methanol solutions onto pre-treated ITO substrates. The thicknesses of interlayers are calculated according to their UV absorption and Beer's law, as described in our previous work (e.g., 0.13 mg/mL gave 1 nm films, 0.25 mg/mL gave ~2 nm films and 0.50 mg/mL gave ~5 nm films, approximately).[3] The conjugated polyelectrolyte interlayer, PPFNBr, was spin casted from its methanol solution (0.13 mg/mL) at 2000 rpm. The neutral star-shaped interfacial material (TrOH) was spin casted from its ethanol solution at 2000 rpm to get a thin layer of nearly 9 nm. All the films were baked at 60°C for 10 min in

air. In all cases, the spin-coated structures were then transferred into a nitrogen-filled glove box (< 0.1 ppm O_2 and H_2O). P3HT:ICBA (1:1 by weight, 250 nm thickness), was then spin-coated on top as per from 44 mg/mL *o*-dichlorobenzene (*o*-DCB) solution at 1000 rpm and annealed at 150°C for 15 min inside the glove box. P3HT:PC71BM (1:0.8 by weight, 90 nm thickness), was then spin-coated on top as per from 18 mg/mL *o*-DCB solution at 1000 rpm and annealed at 100°C for 10 min inside the glove box.

Top electrodes were then thermally evaporated through a shadow mask onto the active layer. The 2.0 nm V_2O_5 followed by 80 nm Ag or 8.0 nm MnO_3 followed by 80 nm Ag top electrodes were thermally deposited in vacuum at a base pressure of 2×10^{-6} Torr. Finally, all the devices were encapsulated in the glove box with the epoxy and hardener (1:1 in volume) mixture and covered with a glass slide. For devices using P3HT:ICBA as the active layer, the active area of the pixels as defined by the overlap of anode and cathode area was 0.0525 cm^2. Eight individual PSC devices were prepared simultaneously on each substrate. For devices with P3HT:PC70BM active layer, the active area of the pixels as defined by the overlap of anode and cathode area was 0.05 cm^2. Four individual PSC devices were prepared simultaneously on each substrate.

The current density-voltage (*J-V*) characteristics were measured using a Keithley 2400 source measure unit. The photocurrent was measured under AM 1.5G illumination (through the glass substrate) at 100 mW/cm^2 using a Newport Thermo Oriel 91192 1000W Solar Simulator. Incident photon conversion efficiency (IPCE) spectra were recorded using the monochromated (Bentham) output from a tungsten halogen lamp calibrated with a Newport UV-181 photodiode; phase sensitive detection with a lock-in amplifier was used to increase signal to noise.

4. Optical and surface properties

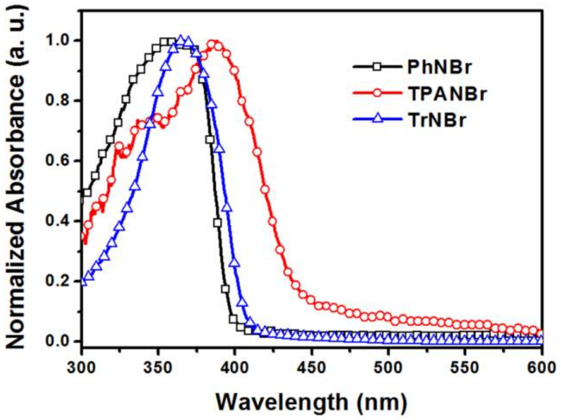

Figure S1. Normalized UV-Visible absorption of PhNBr (black squares), TPANBr (red circles) and TrNBr (blue triangles) in the film state.

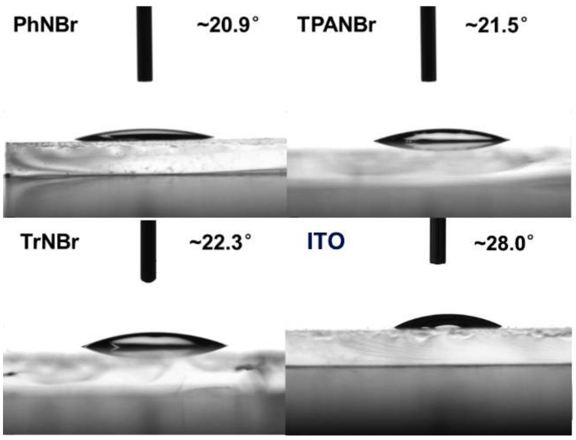

Figure S2. Water contact angle of star-shaped interlayer films. The average thicknesses of all the films are 2 nm (0.25 mg/mL in methanol at 5000 rpm).

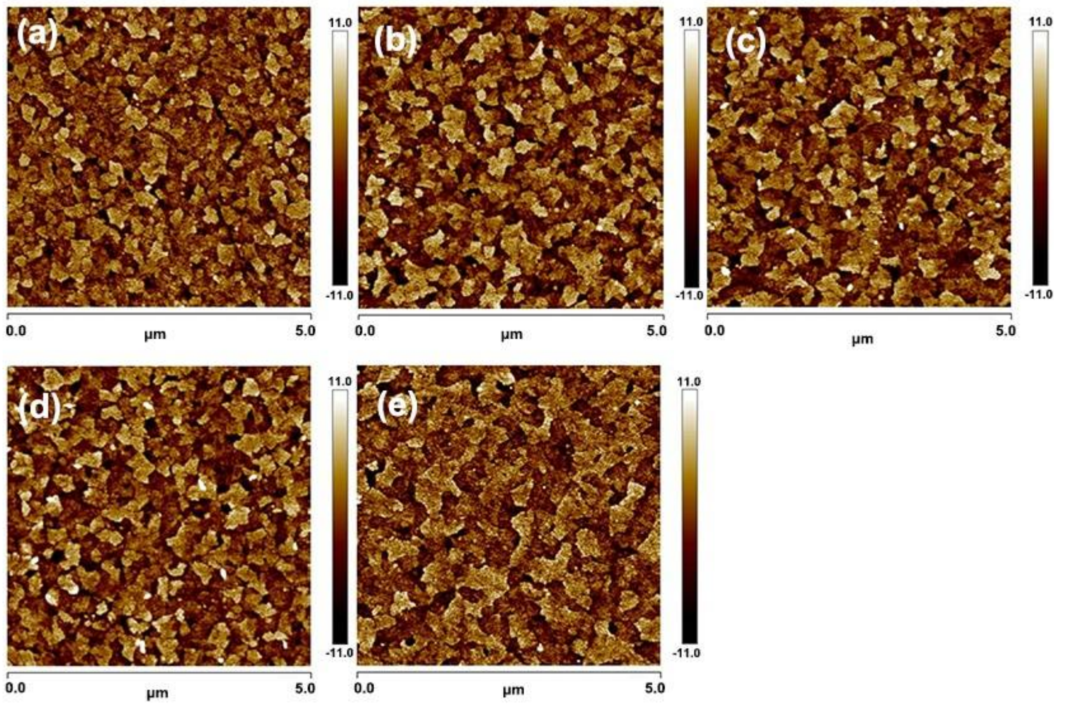

Figure S3. Atomic forced microscopy topography of a) bare ITO b) ITO/PhNBr c) ITO/TPANBr d) ITO/TrNBr e) ITO/PPFNBr. The average thicknesses of all the films are 2 nm (0.25 mg/mL in methanol at 5000 rpm).

5. Detailed device performance and characterization

5.1 Detailed device performance for inverted solar cells with configuration of ITO/PhNBr, TPANBr, or TrNBr/P3HT:ICBA/V_2O_5/Ag

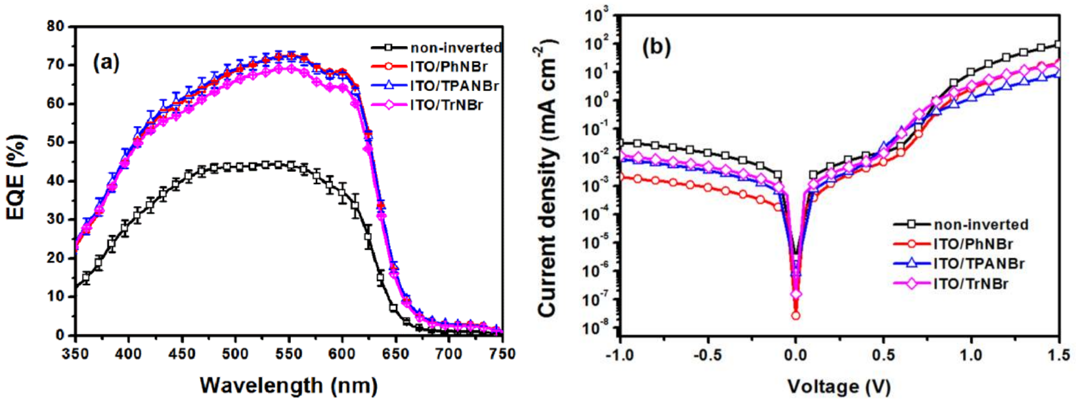

Figure S4. (a) External quantum efficiency spectra and (b) *J-V* characteristics in dark of the reference Ca/Al devices (squares) and the inverted devices with optimized PhNBr (circles), TPANBr (triangles) and TrNBr (diamonds) interlayers. All the inverted devices were measured after reaching maximum PCE values.

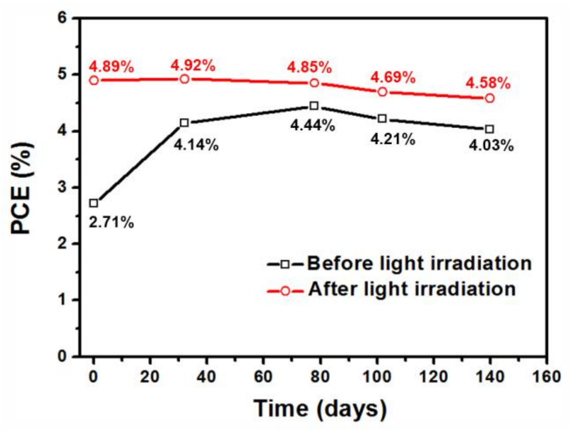

Figure S5. Air stability tests on encapsulated P3HT:ICBA devices with inverted structure with ITO/PhNBr (triangles) as cathode. The PCE values were measured before light irradiation (black squares) and after light irradiation until reaching saturated values (red circles).

5.2 Detailed data for light soaking issues

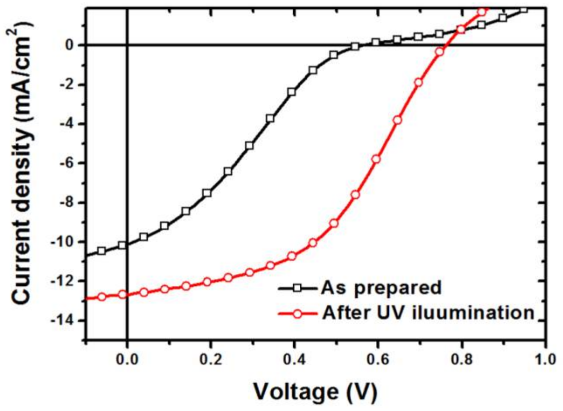

Figure S6. *J-V* characteristics under AM 1.5G irradiation at 100 mW/cm^2 of the inverted devices as prepared (black lines and squares) and after only UV illumination (365 nm) until the saturated value (red lines and circles). The device structure was ITO/PhNBr/P3HT:ICBA/V_2O_5/Ag.

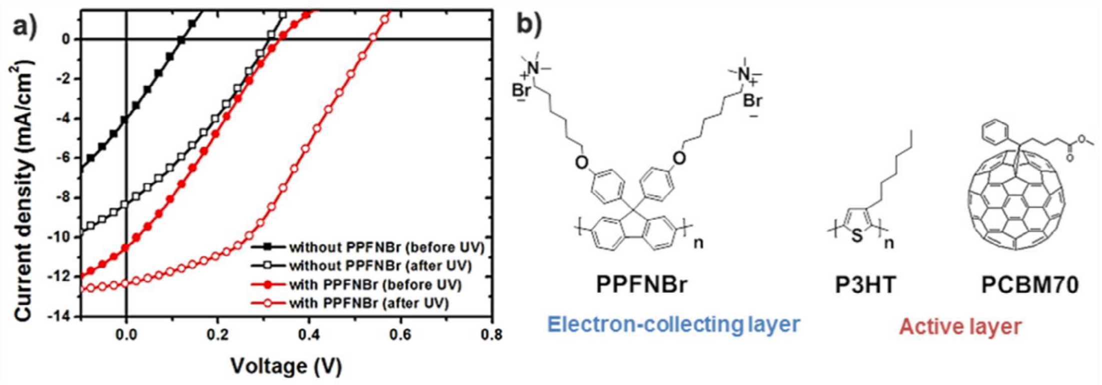

Figure S7. *J*-*V* characteristics under AM 1.5G irradiation at 100 mW/cm^2 of the inverted devices with (red lines and circles) or without (black lines and squares) PPFNBr interlayer before (filled symbols) and after (open symbols) light illumination for 10 min. The device structure was ITO/with or without PPFNBr/P3HT:PC70BM/MoO_3/Ag.

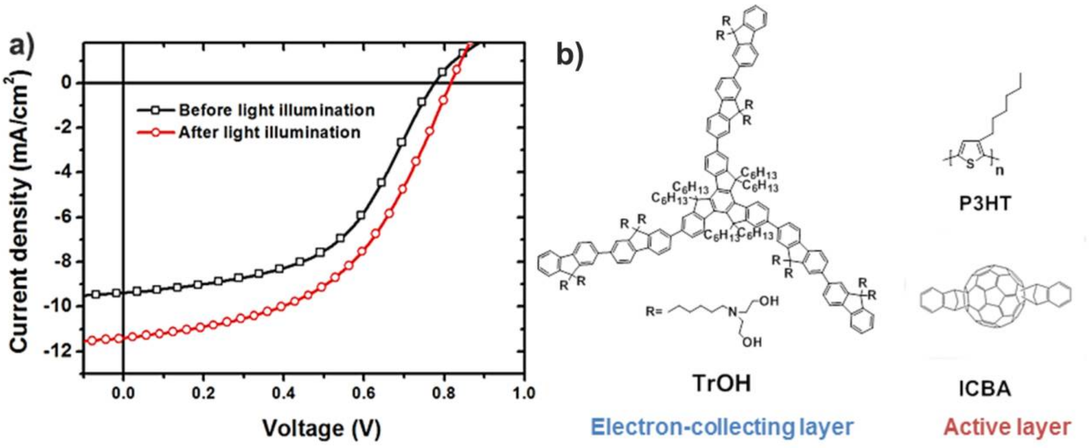

Figure S8. *J*-*V* characteristics under AM 1.5G irradiation at 100 mW/cm^2 of the inverted devices as prepared (black lines and squares) and after UV illumination (red lines and circles). The device structure was ITO/TrOH/P3HT:ICBA/V_2O_5/Ag.

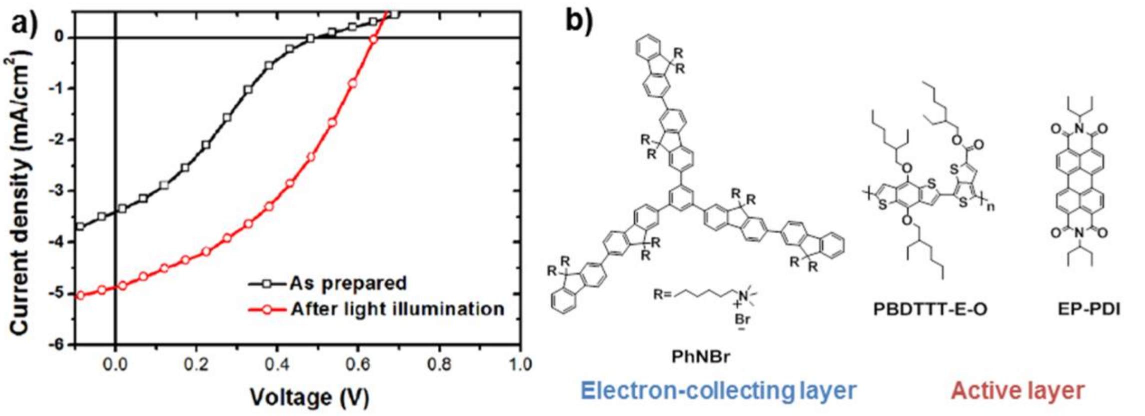

Figure S9 *J-V* characteristics of the inverted solar cells before (black lines and squares) and after (red lines and circles) light illumination were characterized under 0.92 Sun (AM1.5G); The device structure was ITO/PhNBr/PBDTTT-E-O:EP-PDI/V_2O_5/Ag.

Table S1 The electrical parameters of the inverted devices with PhNBr as electron-collecting interlayer before and after light irradiation to the ultimate value (0.92 Sun).

Cathode	V_{OC} (V)	J_{SC} (mA/cm^2)	FF (%)	PCE[a] (%)
As prepared	0.498	3.40	27.8	0.51
After UV illumination	0.640	4.87	40.1	1.36

[a] The devices were characterized under 0.92 Sun (AM 1.5G).

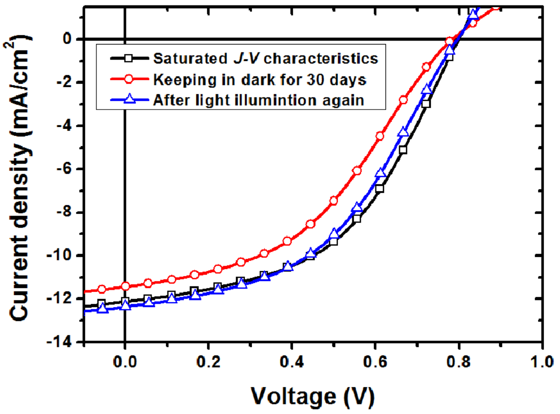

Figure S10. *J-V* characteristics under AM 1.5G irradiation at 100 mW/cm^2 of the inverted devices after light illumination until saturated value (black lines and squares), being kept in dark for another 30 days (red lines and circles) and being illuminated to the saturated PCE

value again (blue lines and triangles). The device structure was ITO/PhNBr/ P3HT:ICBA/V_2O_5/Ag.

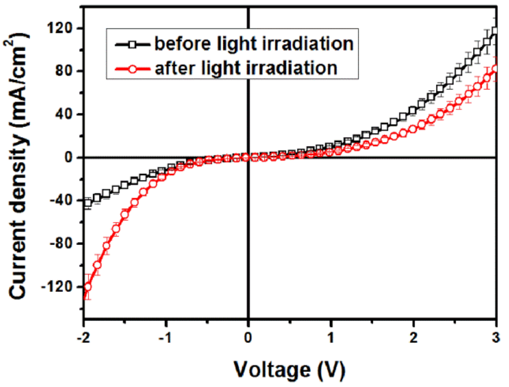

Figure S11. *J-V* characteristics of electron-only device with structure of ITO/PhNBr/ P3HT:ICBA/Ca/Al measured before and after light illumination (10 min) in dark. The voltage was applied to the bottom ITO contact.

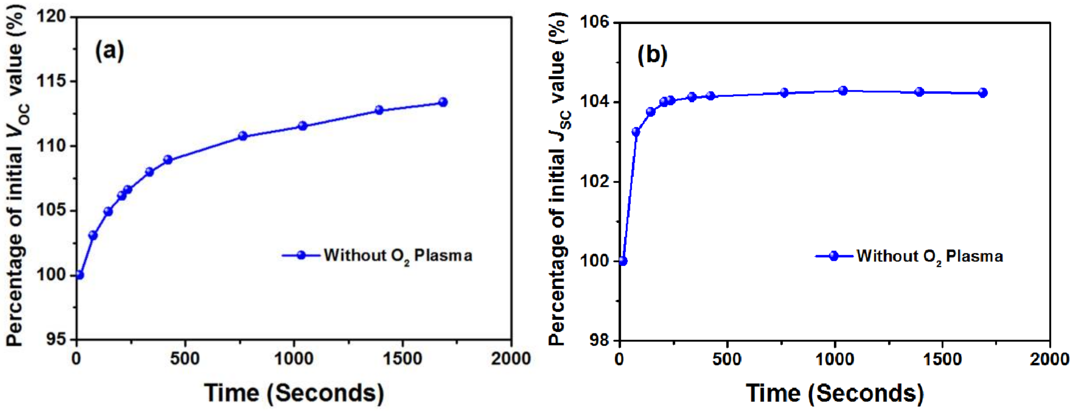

Figure S12. Time-dependent variation of V_{OC} (a) and J_{SC} (b) of PhNBr based inverted devices without oxygen plasma processing.

Reference

[1] W. Xu, X. Zhang, Q. Hu, L. Zhao, X. Teng, W.-Y. Lai, R. Xia, J. Nelson, W. Huang, D. D. C. Bradley, *Org. Electron.* **2014**, 15, 1244.

[2] W. Xu, W.-Y. Lai, Q. Hu, X. Y. Teng, X. W. Zhang, W. Huang, *Polym. Chem.* **2014**, 5, 2942.

[3] W. Xu, Z. Kan, T. L. Ye, L. Zhao, W.-Y. Lai, R. Xia, G. Lanzani, P. E. Keivanidis, W. Huang, *ACS Appl. Mater. Interfaces* **2015**, 7, 452.
